# Single Crystal Organic Nanoflowers

**DOI:** 10.1038/s41598-017-17538-0

**Published:** 2017-12-11

**Authors:** Sajitha Sasidharan, Shyni P. C., Nitin Chaudhary, Vibin Ramakrishnan

**Affiliations:** 1 Molecular Informatics and Design Laboratory, Department of Biosciences and Bioengineering, Indian Institute of Technology Guwahati, Guwahati, 781039 India; 2 Biophysical Chemistry Laboratory, Department of Biosciences and Bioengineering, Indian Institute of Technology Guwahati, Guwahati, 781039 India

## Abstract

Nano-flowers reported so far were mostly constituted of inorganic or hybrid materials. We have synthesized and crystallized a new organic compound, 1, 2-bis(tritylthio)ethane forming an organic nano-flower consisting of single crystalline petals. Crystal structure at nano and micro level indicates that π-π stacking interactions between aromatic systems is the principal factor governing molecular recognition and assembly. Single crystal X-ray Diffraction (S-XRD) supported by Selective Area Electron Diffraction (SAED) experiments indicate the single crystalline nature of the flower-like assembly even at the nanoscale. In order to fabricate the nanoflower as a potential stimulus responsive material; the ‘petals’ were coated with magnetite nanoparticles, verified by Energy-dispersive X-ray spectroscopic (EDX) analysis. Herein, we have further tested the potential utility of the hybrid material in water remediation as a nano-based adsorbent for removal of heavy metals like chromium.

## Introduction

Structural and functional adaptability of organic molecules has always been a source of inspiration to explore the possibility of designing stable, well-defined molecular architectures important for the development of newer technologies. Through this paper, we report synthesis and crystallization of a pure organic molecule and its assembly to a stable crystalline organic nanoflower. We successfully attempted to verify the possibility of converting this nano-assembly to a functional hybrid material by coating it with magnetite. The resulting magnetite hybrid material was examined for their potential utility as a nano-adsorbent for heavy metal removal and groundwater remediation.

Nano-flowers due to their thin and open edges has been a promising candidate fabricated for various important applications such as catalysis, biosensors and optoelectronic devices^1^. The data reported on nano-flowers are either inorganic^1^, or hybrid materials^2,3^. Carbon, elemental metals and compounds of metals with fifth and sixth group elements were the principal constituents of such assemblies. Single crystal organic nanoflowers have not been discovered so far to the best of our knowledge, though, single crystal inorganic nanoflowers^4^ assemblies of organic nanoflowers^5,6^, hybrid organic-inorganic nanoflowers using copper (II) ions^2,7^ diphenylalanine^8^, N,N′-diphenyl-N,N′-bis(1-naphthyl)-1,1′-biphenyl-4,4′-diamine (NPB)^9^ and DNA based nanoflowers have been reported recently^10–12^.

Contamination of water by heavy metals has been a concern for many decades, majorly affecting the densely populated nations^13,14^. Even the presence of very low concentration of heavy metals such as chromium, poses serious threat to human health^15^. Chromium exists as different species among which Cr (III) and Cr (VI) are the most stable ones and the oxidation states of which determines the bioavailability, toxicity, and fate of the metal in the environment. Chromium in the trivalent state is an essential trace nutrient, and it naturally occurs in the environment. Chromium(III) is thermodynamically stable, relatively insoluble, less mobile and relatively nontoxic due to the formation of insoluble oxyhydroxides. In contrast, the toxicity of hexavalent chromium is high due to its strong oxidizing potential and speciation as a weakly sorbing anionic chromate (CrO_4_^2−^ or dichromate (Cr_2_O_7_^2−^). Reduction of Cr(VI) to Cr(III) is an important mechanism for remedying the health hazards caused by chromium contamination. Various methods such as photo-catalytical oxidation, chemical coagulants, electrochemical techniques, bioremediation, ion-exchange, reverse osmosis, and adsorption have been adapted for water treatment^13,16–20^. In comparison to the conventional methods, nano-adsorbents are found to be the more efficient, and magnetic solid-phase extraction has been very effective owing to their capability to get isolated from the sample by using an external magnetic field^21,22^. Magnetite nanoparticles with small size and high surface area, ensures high extraction efficiency within a short period^22^. But the particles with large surface area easily undergo aggregation in the solution which could decrease their efficiency. Most of the magnetite-based nanomaterials used in the adsorption have been surface functionalized or modified^23^, which in turn is a complicated and intensive process. In this study, we present magnetically decorated assemblies of 1, 2-bis(tritylthio)ethane at the nanoscale, generated out of relatively simple procedures, extensively adsorbing heavy metals like Cr(VI) on its surface.

## Results

**Figure 1:**
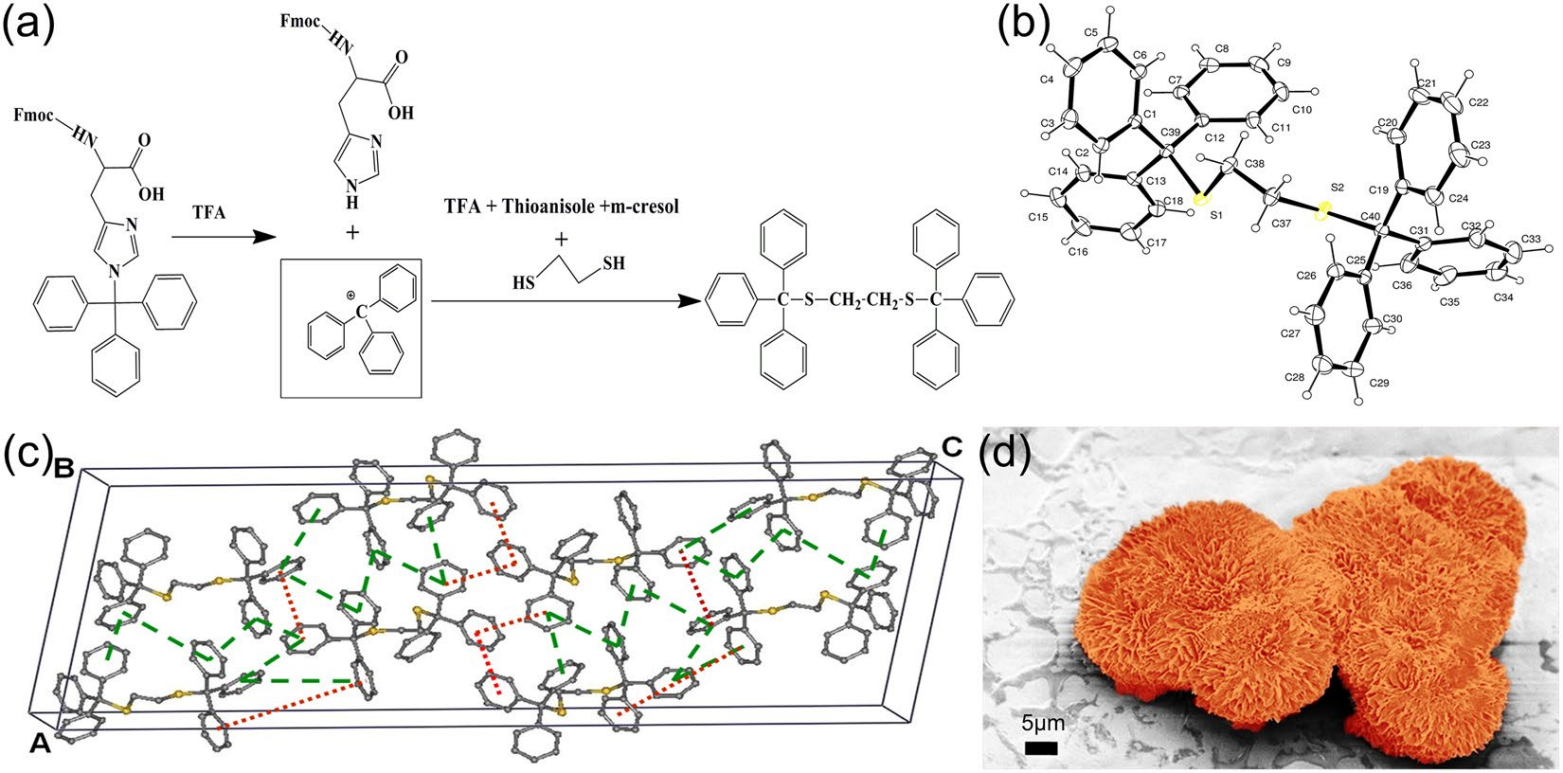
Synthesis, crystallization and nano-assembly of 1, 2-bis(tritylthio)ethane. (**a**) Scheme illustrating the reaction conditions for the synthesis and crystallization of 1, 2-bis(tritylthio)ethane. (**b**) ORTEP diagram of 1,2-bis(tritylthio)ethane. (**c**) The schematic unit cell of 1, 2-bis(tritylthio)ethane, showing the type of interactions in phenyl embraces (The green dashed line indicates T shaped edge to face interaction, and the red dotted line indicates parallel displaced orientation). (**d**) False-colored FE-SEM image showing flower like assembly.

**Figure 2:**
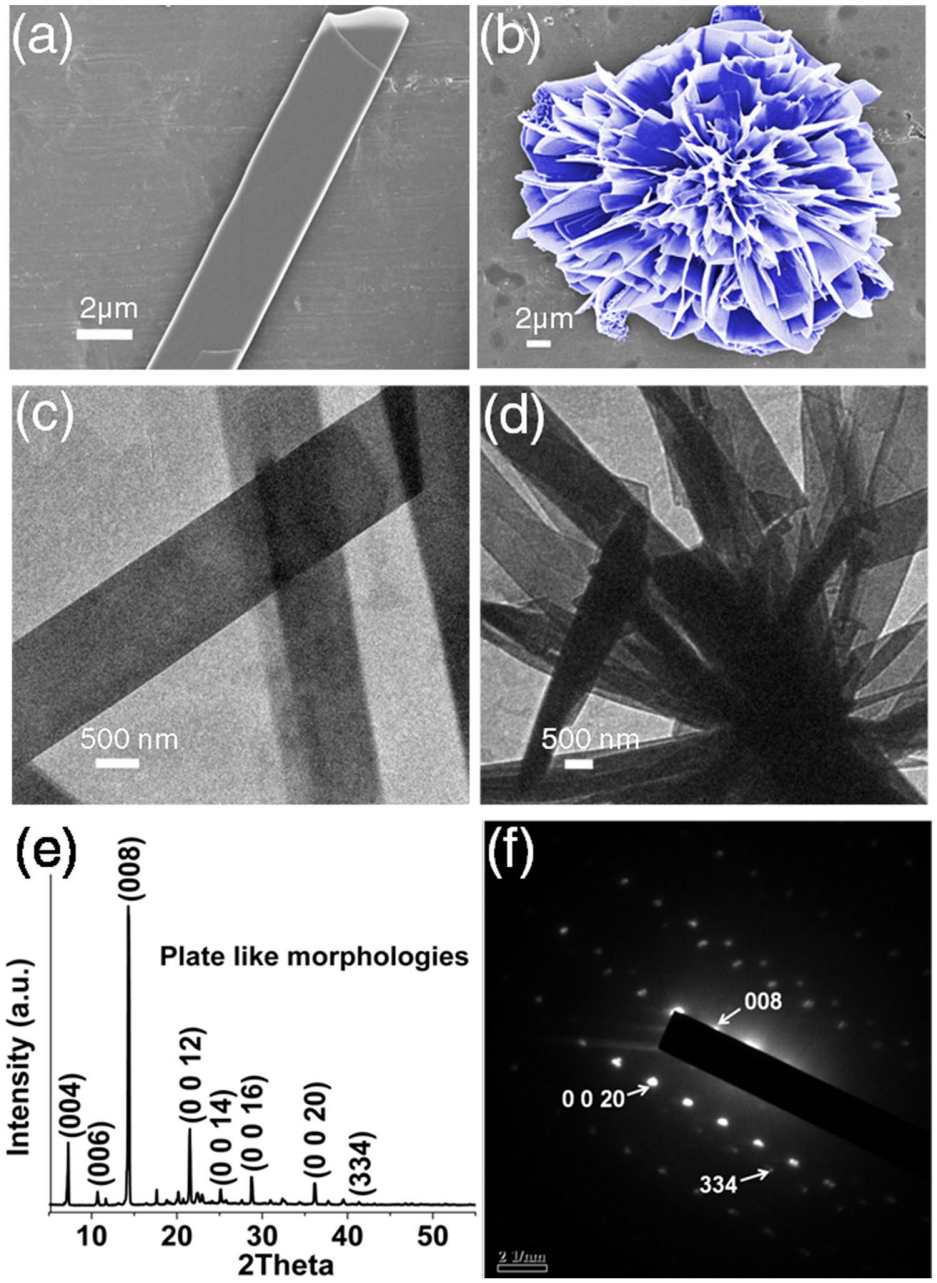
Morphological and structural characterization of 1, 2-bis(tritylthio)ethane assemblies. (**a**,**b**) FE-SEM images of different structural morphologies formed by 1, 2-bis(tritylthio)ethane**;** (**a**) plate-like assemblies and (**b**) nanoflower (False coloured in blue). (**c**,**d**) TEM images showing (**d**) nanoflowers made from (**c**) individual plate-like morphologies. (**e**) P-XRD of plate-like structures. (**f**) SAED pattern of the flower with a diffraction spot indexed as (008), verify the constitution and single crystalline nature of the flower at nano-level.

The molecule we report here is 1, 2-bis(tritylthio)ethane (Fig. 1a). Triphenylmethyl (trityl) group is a stable radical first reported by M. Gomberg in 1900^24^. Tritylcations with a positive charge on the α-Carbon atom is one of the most versatile and stable molecule widely used in biomedical applications such as neurotransmission measurements, oligonucleotide arrays (DNA chips) and as organic dyes^25^. Three aromatic rings stabilize the positive charge on α-Carbon atom by the resonance effect. The acid labile nature of trityl group is utilized in serving as a class of protective group widely used in nucleoside, oligonucleotide, peptide and carbohydrate chemistry^25^. We first observed crystallization of 1, 2-bis(tritylthio)ethane while synthesizing small peptides on solid support. Solid phase peptide synthesis involves sequential coupling of individual amino-acids, with alternate cycles of deprotection and coupling^26^. After completion of synthesis, with Fmoc protocol, acidic reagents like trifluoroacetic acid (TFA) are used to cleave the alkoxy benzene ester group at the linker. Often scavengers like 1, 2-ethanedithiol (EDT) and thioanisole are used to capture the carbocations formed by the cleavage of trityl groups, from side-chain substituents. We first observed the formation of 1, 2-bis(tritylthio)ethane in one such deprotection reaction. 1, 2-bis(tritylthio)ethane was spontaneously formed by treating trityl group released from the side chain of trityl-protected amino acid, with EDT, m-cresol and thioansiole in the presence of TFA (Fig. 1a). Solid state structure of 1, 2-bis(tritylthio)ethane was determined by X-ray crystallography. Single crystal X-ray diffraction (S-XRD) (Mo Kα, λ = 0.71073 Å) analysis details of the obtained crystals were deposited in Cambridge crystallographic database (CCDC deposition number 1412852). The molecule crystallizes in the monoclinic system, with a C2/c space group in a unit cell consisting of 8 molecules. A unit cell containing fragment of crystal structure is presented in Fig. 1c, and the complete details are shown in Table S1. The melting point of the crystals was found to be in the range of 180–185 °C. The purity of synthesized crystals was verified using proton NMR (Figure S1). The morphologies and microstructures of 1, 2-bis(tritylthio)ethane crystals formed in diethyl ether were examined using Field Emission - Scanning Electron Microscope (FE-SEM). FE-SEM analysis shows plate -like morphologies (Fig. 2a) of varying width in nm-μm range, collectively aligned in almost regular petal-like orientation as in a typical nanoflower (Figs 1d,2b). Detailed examination of a single nanoflower shows that they are composed of plate-like and curled thin petals, with smooth surfaces as we normally observe in a natural flower (Figure S2). Morphology evolution of similar kind as a result of collective and cooperative alignment of nanorods^27,28^, nanoblades^29^ and nanosheets^30,31^ has already been reported by various groups with inorganic materials. We verified the chemical composition of both the morphologies, by recording their respective Raman spectra (400–3200 cm^−1^, Figure S3, Table S2). TEM analysis (Fig. 2c,d) further supports the results from FE-SEM and Raman, indicating the assembly of nano-plates to nanoflowers. S-XRD and Powder X-ray diffraction (P-XRD) (Fig. 2e) ensured the crystallinity of the plate-like morphologies, while the Selected Area Electron Diffraction (SAED) pattern (Fig. 2f) indicate the single crystalline nature of the flower-like assembly even at the nano-scale. In addition to this, the indexed peak (008) in the SAED pattern of the single crystalline nanoflower (Fig. 2f) corresponding to the major crystalline phase in the P-XRD of plate-like structures (Fig. 2e) verify that the observed structure is an assembly of individual petals with plate-like morphology. The weak planes in the SAED pattern correspond to the secondary crystalline structures in the nanoflower. Comparison of the experimental powder X-ray diffraction pattern with simulated PXRD pattern of the single crystal has also been performed. As shown in Figure S4, Bragg’s peak positions of the experimental XRD are in good agreement with the simulated pattern.

**Figure 3:**
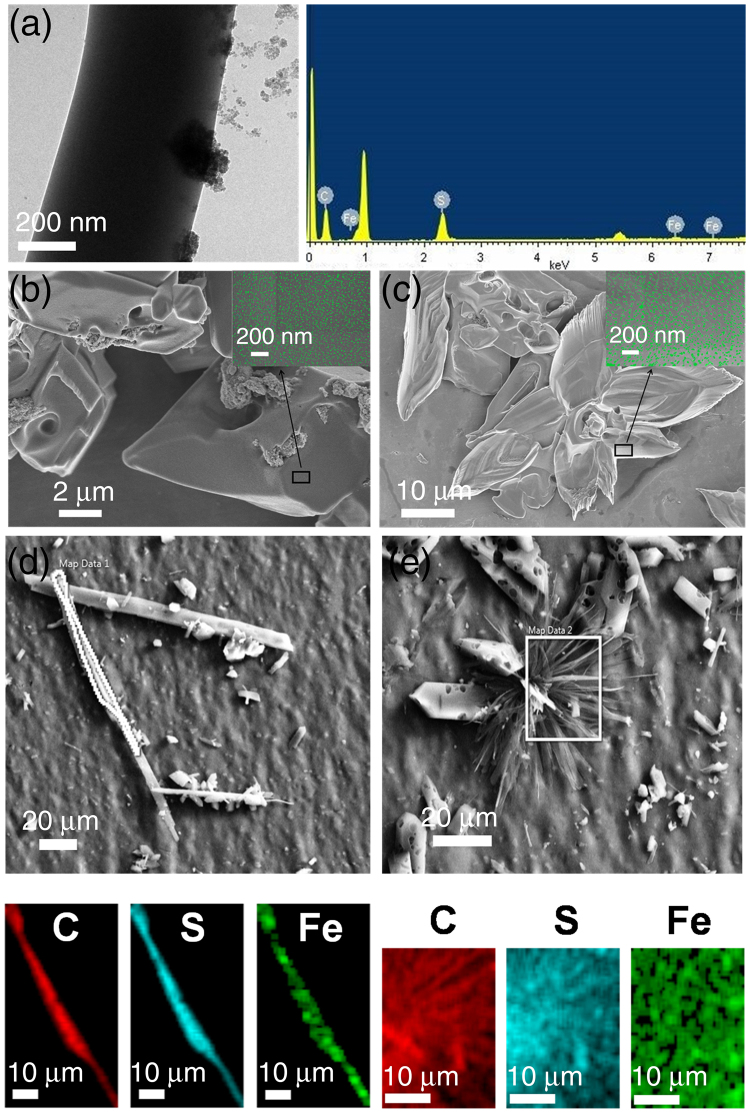
Characterization of magnetite coated 1, 2-bis(tritylthio)ethane assemblies. TEM analysis (**a**) showing the impregnation of magnetite on to the nano-assembled plate-like structure, and its EDX confirming the attachment of magnetite. FE-SEM micrograph of the magnetite coated plate (**b**) and flower-like morphologies (**c**) with insets showing the enlarged view of the coated areas. Magnetite nanoparticles in the insets are false-colored (green) for better understanding. Elemental mapping of the highlighted areas in the nanoassemblies (**d**,**e**) further confirms the dispersion of magnetite on the entire surface.

In the next phase of our study, in order to fabricate the nanoassembly (flower) as stimulus responsive material, the trityl system was coated with the magnetite nanoparticles synthesized using co-precipitation method (Figure S5)^32,33^. This line of enquiry would be particularly interesting in the design and development of organic-inorganic hybrid materials with tunable physical properties. Magnetite (Fe_3_O_4_) was chosen as an oxide of choice, owing to its desirable properties like amphoteric surface activity, easy dispersion ability and high surface to volume ratio^34^. Magnetite as adsorbents finds it its limitations in continuous flow systems since bare magnetite nanoparticles are is highly susceptible to oxidation and tends to agglomerate in aqueous solutions.Stabilization of the nanoparticles is generally attained by surface functionalization^23^. Herein we are showing the adherence of the magnetic material(magnetite) by means of a non- covalent interaction^35^. 1, 2-bis(tritylthio)ethane is grown in a solution of magnetite dispersed in diethyl ether. Adherence of magnetite on the samples is evident from the electron microscopic images. High - Resolution Transmission Electron Microscopic (HR-TEM) analysis with Energy-dispersive X-ray spectroscopy (EDX) (Fig. 3a) reveals the attachment of the synthesized magnetite to the plate-like assemblies. FE-SEM analysis (Fig. 3b,c) of both the plate and flower-like morphologies show the distribution of coated material on the surface. Dispersion of magnetite on the entire surface of the plate and flower-like morphologies is further verified by elemental mapping via energy-dispersive X-ray spectroscopy (EDS) mapping (Fig. 3d,e), where, Fe is found to be distributed over the entire surface, demonstrating the successful impregnation of metals resulting in an organic-inorganic hybrid nanoflower.

**Figure 4:**
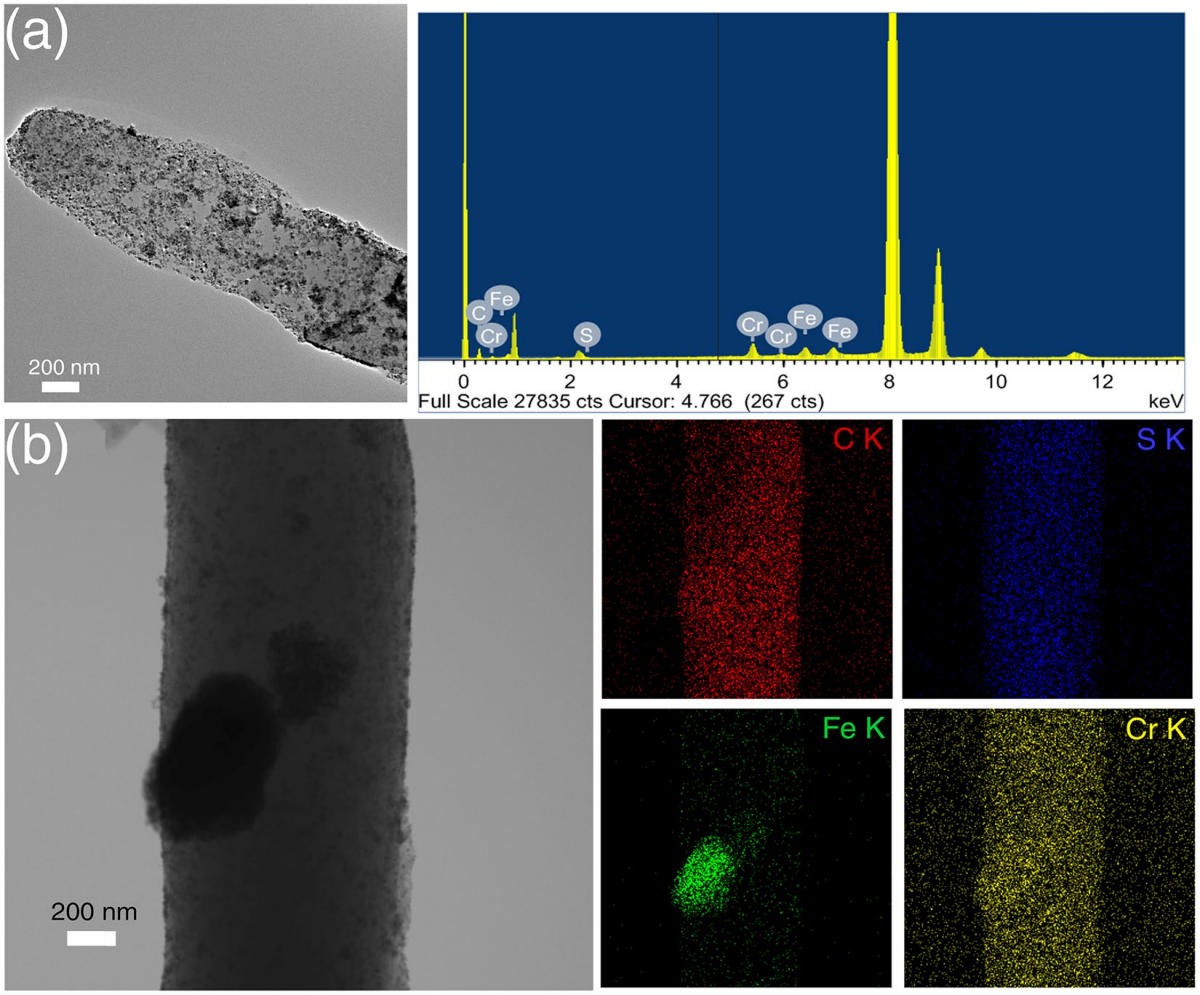
Elemental distribution on the surface of nanoassemblies probed by EDS-mapping. TEM and EDX spectra of chromium (Cr) sequestered hybrid material (**a**). Backscattered electron image shown in the top left panel of figure b, to verify elemental composition. Elemental spot maps of C, S, Fe and Cr, measured by STEM-EDX, are shown in the following panels of (**b**), confirming the Cr sequestration on to the hybrid nano assembly.

In the following experiment, we tested whether the stimulus responsive nature of the magnetite coated hybrid material synthesized in this work can be employed in water remediation by adsorbing heavy metal (Chromium).The hybrid nano-assemblies containing magnetite were continuously vortexed in the chromium solution. After 24 hours of continuous vortexing, the samples were analyzed for adsorption of chromium. Field Emission Transmission Electron Microscope (FE-TEM) analysis and EDX spectra (Fig. 4a) of the sample confirm the presence of Cr getting attached on to the surface. STEM-EDS mapping was performed to further confirm the adsorption of minerals. Elemental mappings for C, S, Fe and Cr on the hybrid material are shown in Fig. 4b. The plate-like architectures providing large surface area for the adsorption of magnetite also exhibited higher specificity towards Cr metal removal. The result of STEM EDS-mapping shown in Fig. 4 reveals the distribution of carbon and sulfur in 1,2 -bis (tritylthio) ethane, with Fe, and Cr sequestrated on its surface. Through this experiment, we intend to demonstrate the potential of a pure organic nanoflower, in acting as a nano-adsorbent for water remediation.

## Discussion

The challenge to generate and perfect nano and micro level stable molecular architectures through self-assembly lies in two mutually fulfilling exercises; i) synthesis of individual building blocks with the right composition and ii) design and fabrication of assembled architectures^36,37^. The single crystal organic compound we report here assembles to a flower- like morphology, by means of a supra-molecular interaction network, thus becoming one among the rare organic nanoflowers reported so far. Multiple phenyl embraces is an important feature of molecules containing the fragment XPh_y_ (X is any tetrahedral atom and y = 2, 3, 4) while they engage each other through a supramolecular interaction network. 1, 2-bis(tritylthio)ethane molecule has six phenyl rings, which can form multiple phenyl embraces (MPE). Such orthogonal (edge to face, or *T shaped*) and parallel (offset face to face or parallel displaced, *PD*) phenyl embraces were earlier identified in crystals^38^. Phenyl embraces, both T and PD shaped in 1, 2-bis(tritylthio)ethane crystal structure are shown in Fig. 1b. The green dashed-lines indicate T shaped edge to face interaction, and red dotted lines indicate parallel displaced orientation. The entire assembly is a result of well-organized repetitive patterning of T shaped and PD interactions. Such face to face or edge to face quadrupole stacking between benzyl side-chain groups account for molecular recognition process in various fibrillar and nanotube formation involving dipeptides with aromatic side chains are reported^39,40^. The nano-level assembly forming plate-like structures and their co-assembly forming nanoflowers may also be attributed to the attractive interactions between aromatic systems. The distance between typical T shaped phenyl embraces are 4.06 and 7.08 Å respectively between the closest and farthest carbon atoms. This is in good agreement with the earlier reported distance range and energy estimates in the case of Benzene dimer^41^.

We could successfully further demonstrate the adaptability of this organic nanoflower, to an organic-inorganic hybrid material. In comparison to the otherwise cumbersome procedures of magnetite impregnation, formation of hybrid material of 1, 2-bis(tritylthio)ethane with magnetite is a simple one-step procedure. Furthermore, the presented methodology detailing the procedure for removal of a typical heavy metal reported here is expected to be an efficient, cost-effective and environment-friendly approach, owing to the large surface area of both magnetite and the crystalline nano-assemblies. Moreover, magnetite synthesized by different methods has been found effective in removing heavy metals such as As(III), As(V), Cu(II), Zn(II), Pb(II), Hg(II), Ni(II), Co(II), Cu(II) and Cd(II)^42^. Therefore this hybridized material can in principle be fabricated as a tool for the removal of other metals in a future work. Overall, this work presents the potential utility of stable tritylthioethane derivatives, and it’s usage in the design of nanomaterials, where molecular recognition is primarily mediated through aromatic π-π interactions. The ability to externally control the properties of magnetic materials would be highly desirable from fundamental and technological point of view, particularly in view of recent developments in magnetoelectronics, spintronics and environmental applications^43^.

## Materials

All the chemicals and solvents used for experiments are of reagent grade. Amino acid Fmoc-His(trt)-OH and solvents Trifluoroacetic acid, Thioanisole, and 1,2-ethanedithiol were purchased from Sigma-Aldrich. Diethyl ether, m-cresol, Iron (II) sulfate heptahydrate and ferric nitrate were purchased from Merck. Sodium dichromate dihydrate was purchased from Sisco Research Laboratories Pvt. Ltd

## Methods

### Synthesis and crystallization of 1,2-bis(tritylthio)ethane

The compound was synthesized by treating trityl group released from the side chain of Fmoc-His(trt)-OH, with 1,2-Ethane dithiol, m-cresol and Thioansiole in the presence of trifluoroacteic acid(1:2:2:20). Crystallization and purification were done using diethyl ether as a solvent. Melting points were recorded on a Stuart smp30 melting point apparatus.

### *S*ynthesis and coating of magnetite on the assemblies

Magnetite was synthesized by co-precipitation method. Co-Precipitation was done in an alkaline medium by mixing aqueous solutions of FeSO_4_.7H_2_O and Fe(NO_3_)_3_. An anionic surfactant was added for stabilization, and the final mixture was heated at 75 °C. Magnetite obtained was washed with distilled water and dried at 60 °C. Obtained magnetite was dispersed at a concentration of 0.214% in ethyl ether. To the same 1,2-bis(tritylthio)ethane was added at a concentration of 2 mg/ml. On crystallization, the samples were characterized using TEM-EDX for elemental analysis. FESEM and EDX mapping were done using JEOL transmission electron microscope, Model: JEM 2100, for showing the distribution of magnetite on the sample surface.

### Adsorption Studies

Stock concentration for the adsorption studies was prepared by dissolving Na_2_Cr_2_O_7_ at a concentration of 50 mg/ml in dd.H_2_O. Adsorption experiments were conducted at a concentration of 1 mg/ml. Samples were vortexed continuously, and the samples were loaded for analysis after 24 h.

## Characterization

### NMR analysis

^1^H NMR was recorded on a Bruker 600 MHz NMR spectrometer. NMR spectra were recorded in deuterated chloroform (CDCl_3_), and the chemical shifts were reported relative to TMS (δ0 ppm) as an internal reference. Spin multiplicity was abbreviated as follows: s = singlet, d = doublet and t = triplet. ^1^H NMR (600 MHz, CDCl_3_) δ (ppm): 7.32–7.31 (d, 12 H), 7.25–7.22 (t, 12 H), 7.20–7.17 (t, 6 H), 2.1 (s, 4 H).

### X-ray Diffraction

Single crystal XRD measurement of the plate-like crystal was done on a Bruker APEX-II CCD diffractometer with graphite-monochromatized (MoKα = 0.71073 Å) radiation at room temperature [296(2) K]. The X-ray data collection was monitored by SMART program (Bruker, 2003)^44^. Powder XRD data measurements were made using a high power (18 kW) Rigaku TTRAX III X-Ray Diffractometer. The powdered crystals were subjected to X-ray radiation of Cu/Kα with a wavelength of 1.54056 Å. The diffraction patterns were recorded over 2 theta values ranging from 5 to 55° in increments of 0.02 degrees.

### Electron microscopic characterization

Ethyl ether was added to the plate-like crystals and vortexed to yield flower-like morphologies. 20 µl of the sample was loaded for Field-Emission Scanning Electron Microscope (FESEM)analysis, and the morphology of nano-assemblies was analyzed using Zeiss (Model: Sigma) Field-Emission Scanning Electron Microscope at 3 kV. Samples were coated with gold for enhancing the conductivity. 10 μl of the vortexed sample was placed on the 300 mesh copper grid covered with strong carbon film; SAED and Transmission Electron Microscopic (TEM) analysis of the sample was performed on JEOL transmission electron microscope, Model: JEM 2100, operating at 200 kV.10 μl of the hybrid material vortexed with the chromium solution was loaded on the carbon coated grid for analysis. FE-TEM analysis and STEM-EDS mapping were done using JEOL JEM 2100 F.

### Raman Spectroscopy

Raman spectra were recorded using Horiba JobinVyon, Laser Micro Raman System.

## Electronic supplementary material

Supplementary Information

Supplementary Dataset 1
