# Supplementary material for: Single Crystal Organic Nanoflowers
Source: Sci Rep. 2017 Dec 11;7:17335. doi: 10.1038/s41598-017-17538-0 (PMC5725578; doi:10.1038/s41598-017-17538-0)
Supplement: Supplementary file 1 — Supplementary Information [file 41598_2017_17538_MOESM1_ESM.doc]

Single Crystal Organic Nanoflowers

*Sajitha Sasidharan.^†^ Shyni P.C., ^§^ Nitin Chaudhary,^§^ and Vibin Ramakrishnan^†^,**

^†^Molecular Informatics and Design Laboratory, Department of Biosciences and Bioengineering, Indian Institute of Technology Guwahati, Guwahati -781039, India

^§^Biophysical Chemistry Laboratory, Department of Biosciences and Bioengineering, Indian Institute of Technology Guwahati, Guwahati -781039, India

| **Section No.** | **Table of contents** | **Page No.** |
| --- | --- | --- |
| 1 | Crystallography | S3 |
| 2 | ^1^H NMR spectra | S4 |
| 3 | FESEM | S4 |
| 4 | Raman spectroscopy | S5 |
| 5 | P-XRD | S6 |
| 6 | Synthesis of magnetite | S7 |
| 7 | References | S7 |

**Section 1.** Crystallography

**Table S1. Crystal data and structure refinement for 1,2-bis(tritylthio)ethane**

Identification code 1,2-bis(tritylthio)ethane

Empirical formula C_40_ H_34_ S_2_

Formula weight 578.79

Temperature 296(2) K

Wavelength 0.71073 Å

Crystal system, space group Monoclinic, C2/c

Unit cell dimensions a = 16.9966(4) Å alpha = 90 deg.

b = 7.3711(2) Å beta = 99.0830(10) deg.

c = 50.3919(11) Å gamma = 90 deg.

Volume 6234.1(3) Å^3^

Z, Calculated density 8, 1.233 Mg/m^3^

Absorption coefficient 0.198 mm^-1^

F(000) 2448

Crystal size 0.25 x 0.15 x 0.15 mm

Theta range for data collection 0.82 to 25.00 deg.

Limiting indices -18<=h<=18, -7<=k<=7, -53<=l<=53

Reflections collected / unique 34324 / 3967 [R(int) = 0.0391]

Completeness to theta = 25.00 99.8 %

Absorption correction Semi-empirical from equivalents

Max. and min. transmission 0.9709 and 0.9521

Refinement method Full-matrix least-squares on F^2^

Data / restraints / parameters 3967 / 0 / 379

Goodness-of-fit on F^2^ 1.155

Final R indices [I>2sigma (I)] R1 = 0.0359, wR2 = 0.0916

R indices (all data) R1 = 0.0494, wR2 = 0.1089

Largest diff. peak and hole 0.202 and -0.226 e.Å^-3^

**Section 2** NMR


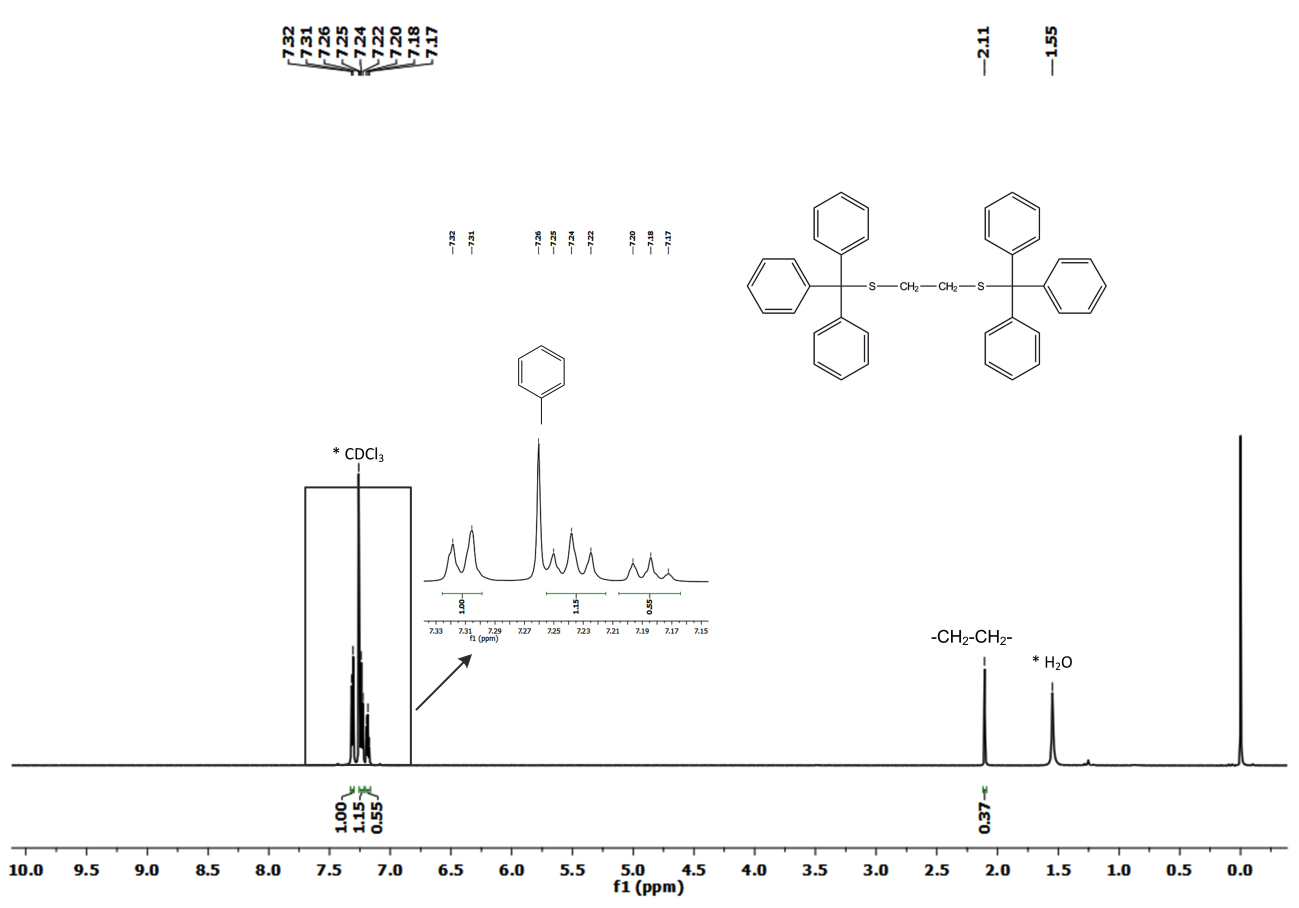


Figure S1. ^1^H NMR of 1, 2-bis(tritylthio)ethane (CDCl_3_, 600MHz)

**Section 3.** FESEM


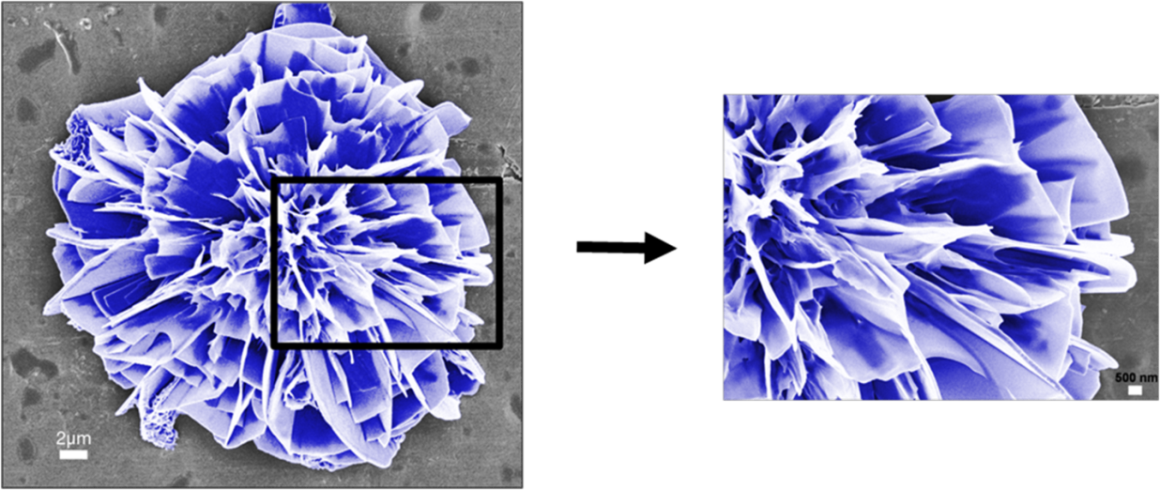
**Figure S2.** Detailed analysis of nanoflower like morphologies showing curled and plate like petals.


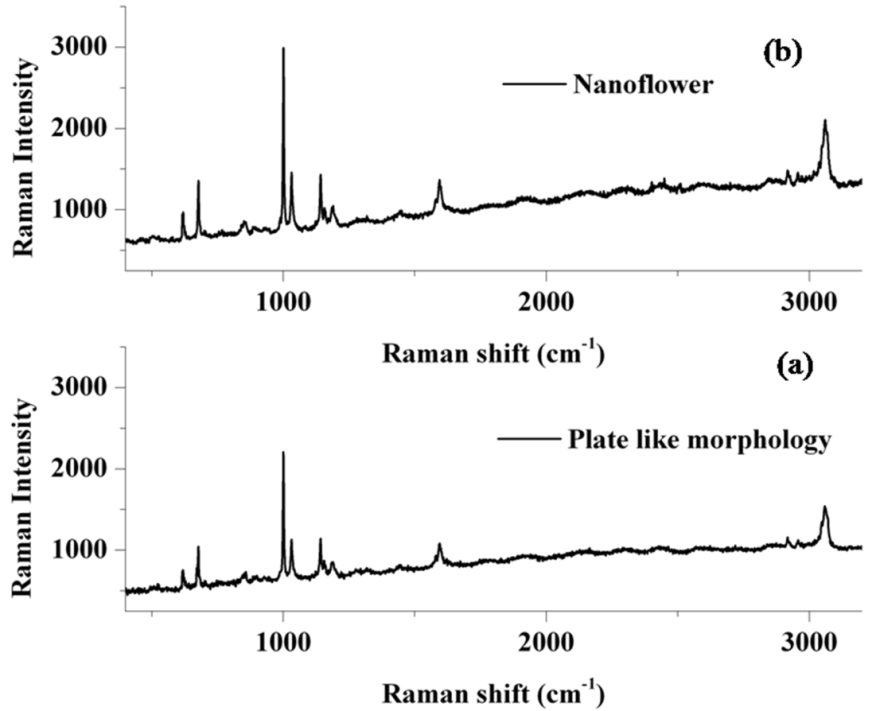
**Section 4.** Raman spectroscopy

**Figure S3.** Characteristic Raman spectra of **(a)** Plate like morphologies, **(b)** nanoflower like morphologies.

**Table S2.** Raman peak assignment for 1,2-bis(tritylthio)ethane

| **S.No** | **Sample Peaks (cm^-1^)** | **Peak assignment** |
| --- | --- | --- |
| 1 | 618.878 – 685.272 | C- S stretch^1^ |
| 2 | 1003.11, 1034.69, 1579.86, 1595.65 | Benzene ring^2^ |
| 3 | 1160.32 – 1195.46 | CH_2_ wag and twist |
| 4 | 3064.46 | CH stretch in aromatic compounds |

Raman spectra observed for both the morphologies (Fig. S2 a & b) appears to be the same, justifying that the plate like morphologies collectively aligns to form the nanoflower like morphology. Also the peak assignment (Table S2) agrees well with the functional groups present in the structure.

**Section 5**. X-ray Diffractogram


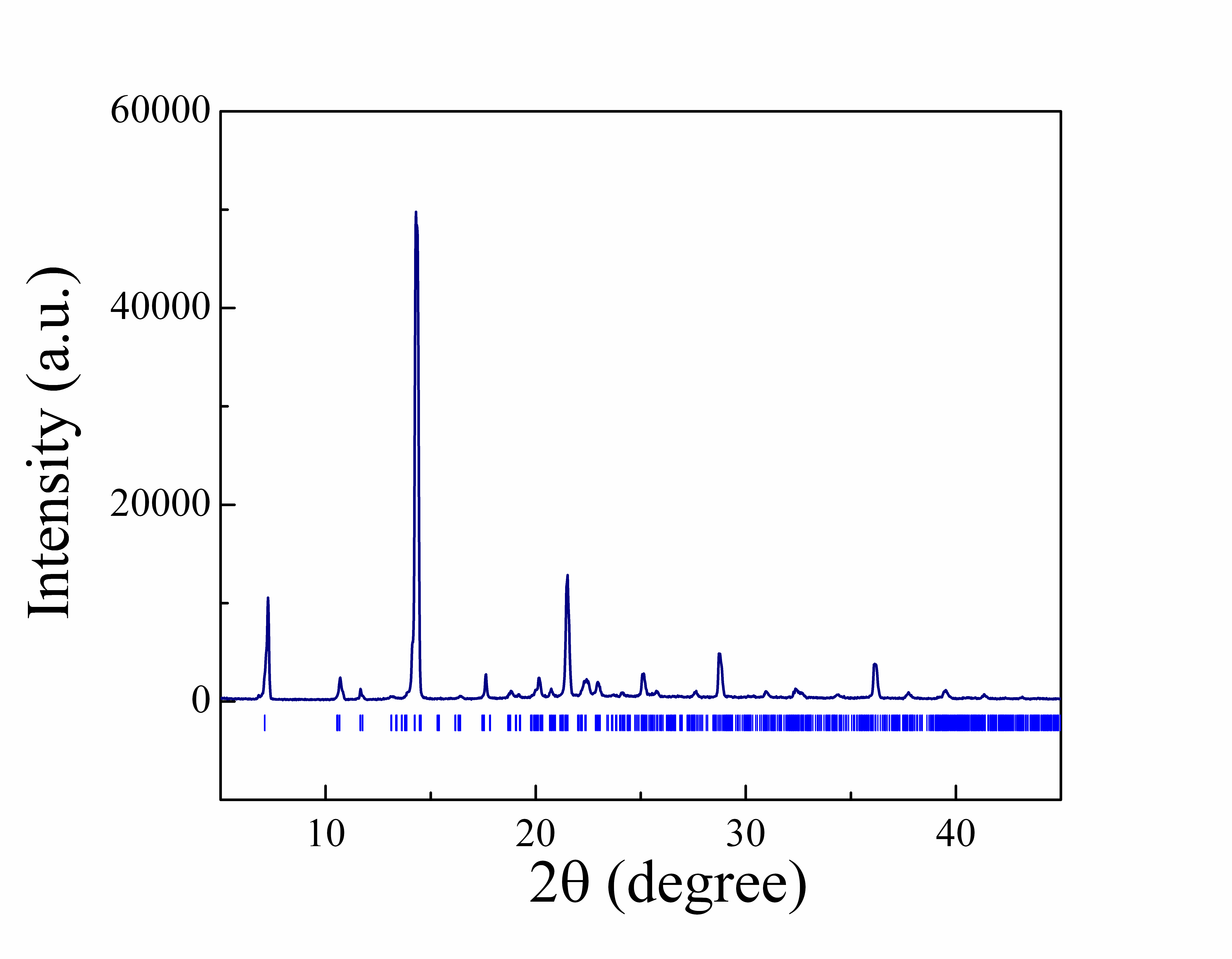


**Figure S4**. A comparison of the experimental powder X-ray diffraction pattern and simulated P-XRD pattern., where the simulated pattern is represented by blue color vertical straight lines

**Section 6. Synthesis of Magnetite**


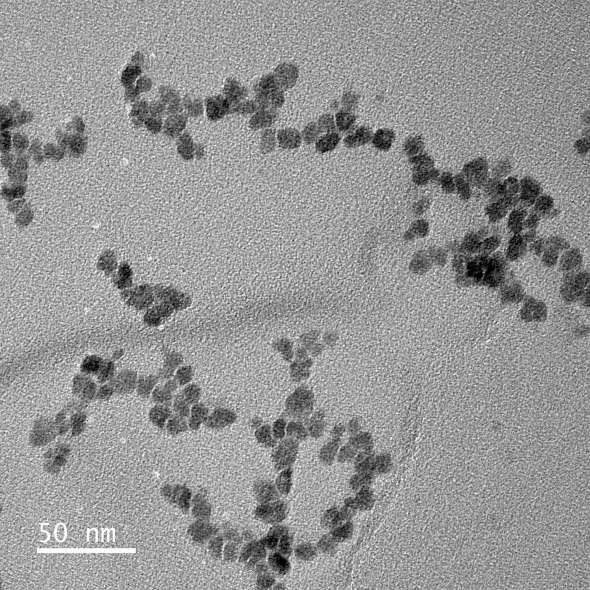


**Figure S5**. Transmission electron micrographs of magnetites synthesized using co-precipitation method

**Section 7.** References

1. Lambert, J.; Shurvell, H.; Lightner, D.; Cooks, R., Organic Structural Spectroscopy Prentice-Hall. *Englewood Cliffs, NJ* **1998**, 31.

2. Lekprasert, B.; Korolkov, V.; Falamas, A.; Chis, V.; Roberts, C. J.; Tendler, S. J.; Notingher, I. *Biomacromolecules* **2012,** *13*, 2181.
